# Neonatal Appendicitis (Part 2): A Review of 24 cases with Inguinoscrotal Manifestation

**Published:** 2015-04-01

**Authors:** V Raveenthiran

**Affiliations:** M.Ch, FRCS (Glas)Department of Pediatric Surgery, Sri Ramasamy Memorial (SRM) Medical College SRM University, Chennai 603203, India

*(Athena stands for abbreviation of Abstracting and Thoughtful Evaluation of Neonatal Articles; but it is also personified by the contributor. Like the Athena of Greek mythology, “she” distills wisdom from published literature)*

**Introduction**

The first successful appendicectomy is often credited to Lawson Tait (1880). But it was Claudius Amyand who, in 1735, successfully resected a perforated appendix from the hernia sac of an 11-year-old boy. In his honor, inguinal hernia containing vermiform appendix is named as Amyand hernia.[1,2] Mucus accumulation and ischemia of the entrapped appendix may precipitate infection and inflammation. In fact, Amyand’s patient presented with fecal fistula of scrotum due to ignored appendicitis. Athena is surprised that even after 280 years such neglected presentation still exists. In 2014 Panagidis et al [3] reported a 25-day-old newborn with fecal discharge through the right scrotum following ruptured appendix in the hernial sac. Absorbed by the inordinate delay Athena looked closely into inguinoscrotal appendicitis of newborn. 

**Hypothesis**

 From her previous study [4] Athena is aware of the disturbingly high mortality of 23% in neonatal appendicitis. This is commonly attributed to diagnostic difficulties and the consequent therapeutic delays. Inflammed appendix in hernial sac, by virtue of its easy accessibility, should not be a diagnostic challenge. Therefore, early diagnosis and zero delay in treatment are expected to result in better outcome. On the other hand, strangulation of the herniated appendix may be associated with more frequent perforations due to ischemic gangrene. Athena is also curious to know if the scrotal disease manifests differently to cause diagnostic confusions. In order to test these hypotheses, she critically reviewed the literature.

**The game plan**

Athena restricted her analysis to neonatal literature published in the last 25 years (1990 - 2015). She defined “neonate” as an infant within 28 days of birth with appropriate age adjustment in case of preterm babies. She searched Pubmed, Embase, AJOL and Indmed using the keyword combination of neonate, newborn, appendix and Amyand’s hernia. She excluded 8 cases of healthy appendices incidentally found in hernial sac, [5,6,7] one case reported in non-English language [8] and one case with insufficient details. [9] Finally, she culled 24 cases suitable for analysis.[3,10-29] 

**Classification**

Losanoff and Basson [30] classified Amyand hernia in adults into 4 subtypes. (Table 1) This system was basically designed to clarify the appropriateness of mesh hernioplasty in the presence of infection. This categorization is inapplicable to neonates because it is often difficult to define in this age group whether sepsis is confined to the hernial sac or not. In fact, workup for neonatal sepsis led to the discovery of hernial appendicitis in 3 cases. [11,15, 29]. Athena tends to classify them as type O (occult). For the same reason, Athena is inclined to base the class definition on localization of clinical manifestation rather than on the extent of sepsis. [Table 1] In her analysis, type 1 Amyand hernia was seen in 8 cases (25%), type 2 in 17 (53%), type 3 in 4 (13%) and type O in 3 (9%). None of the neonates had colo-ileal co-morbidity (type 4) such as Hirschsprung disease, cystic fibrosis or necrotizing enterocolitis (NEC). This is in agreement with Athena’s previous finding that such co-morbidities are not etiologically associated with abdominal appendicitis in newborn.[4]

**Figure:**
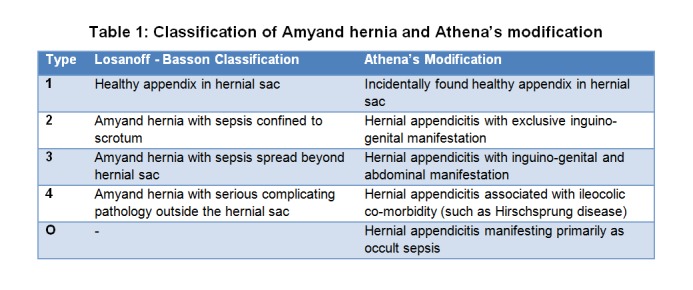
Table 1: Classification of Amyand hernia and Athena’s modification

**Clinical Features**

Table 2 summarizes the differences between abdominal and hernial appendicitis. Both of them are commonly reported from India and Turkey. Striking male predominance of scrotal appendicitis seems to be due to the high incidence of inguinal hernia in boys. As compared to abdominal appendicitis, inguinogenital form occurs in slightly older neonates and it is more common in preterm and small-for-gestational-age babies. These newborn are relatively more active and continue to feed well despite ongoing appendicitis. Therefore, dehydration and circulatory stress such as tachycardia are comparatively less frequent in them. Deceptive bright-look of the infants probably deters early diagnosis. Delay between the onset of symptoms and therapeutic intervention is apparently similar between abdominal and inguinoscrotal disease. However, if the 3 outliers are excluded from the later group, the mean delay falls to 2.1 days. Interestingly, despite early diagnosis the rate of perforation remains as high as 50% in scrotal disease. Notwithstanding this there was no mortality in this group. These paradoxical data suggest that perforation of hernial appendicitis occurs independently of diagnostic delay but its adverse effects are offset by early intervention. This observation is consistent with Athena’s previous conclusion regarding abdominal appendicitis.[4] 

**Figure:**
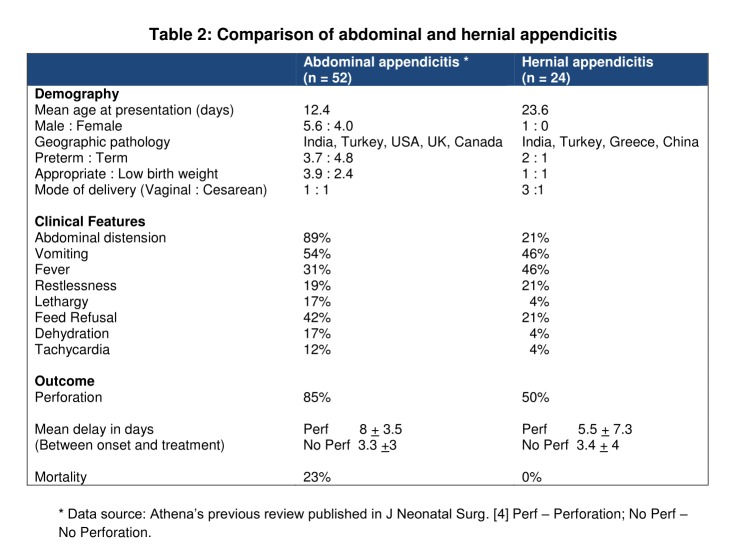
Table 2: Comparison of abdominal and hernial appendicitis

**Risk Factors**

Only 33% of neonates with abdominal appendicitis had birth asphyxia and 44% of them had one or more risk factors of NEC.[4] Among the neonates with hernial appendicitis birth asphyxia was noted in 4 of the 6 (67%) and NEC risk factors in none. However, missing data for a large number of patients precludes any meaningful conclusion. 

**Making a mistaken diagnosis**

 Genital swelling and redness or scrotal pain was noted in all the neonates. Despite these localizing signs and easy accessibility of hernial appendix, correct preoperative diagnosis was not made in any of them. Strangulated hernia (54%), epididymo-orchitis (21%) and testicular torsion (13%) were the 3 commonly mistaken diagnoses. Although ultrasonography excluded torsion by demonstrating good vascular flow in the testis, visualized bowel loops led to misdiagnosis of strangulated hernia. Fortunately, most of the differential diagnosis, by virtue of their own merits, indicated urgent surgical exploration and hence there was no undue therapeutic delay. One newborn [23] was treated with antibiotics with a mistaken diagnosis of orchitis and this caused a delay of 12 days in performing appendicectomy. Imaging studies did not contribute significantly to the preoperative diagnosis. Sepsis screening was positive only in 5 out of 8 cases (63%).[3,10,15,18,21] Importantly, in 3 of them, features of sepsis preceded local symptoms. 

**Surgical access and procedure**

 The choice of surgical incision appears to be influenced by the preoperative diagnosis. Suspicion of strangulated hernia prompted inguinal or inguinoscrotal incision (50%) while a diagnosis of testicular torsion predisposed to scrotal incision (17%). In 3 cases a groin incision was used despite suspecting torsion [14] or orchitis [18,28]. Initial inguinal incision was supplemented with abdominal incision in 2 cases [17,19] while inguinal incision was similarly added to scrotal incision in another 2 cases.[20,24] Laparoscopic approach was used in 2 cases.[16,23] In adult literature there is a debate as to whether hernia repair can be done in the presence of sepsis.[1,2] Disregarding this controversy, combined appendicectomy and hernia repair is the most popular approach (75%) in neonates. In one case [14] orchidopexy was also added for associated undescended testis. However, Athena would not make specific recommendations because long term follow-up is uniformly missing in all the reports. At least in one case [11] the hernia recurred within 6 weeks. Historically also, Claudius Amyand combined appendicectomy and hernia repair in his patient and the hernia promptly recurred. [1,2]

**Testicular outcome**

 Presence of fulminant local sepsis may theoretically induce thrombosis of the delicate testicular vessels. Testicular damage is a potential threat in hernial appendicitis. But Athena is pleased to note 9 out of 10 had viable, albeit congested, testis. Even the sole dusky gonad was salvaged.[29] Athena confidently concludes that testicular viability is not affected by the proximity of perforated appendix. 

**Has it changed over time?**

 In her previous analysis Athena [4] showed that the pattern of abdominal appendicitis in neonates has changed over the period of 100 years. Curious of knowing whether such a change has occurred in hernial appendicitis Athena compared her spreadsheet with the published data of Karaman et.al. [13] (Table 3) A 3-fold increase of reports in the recent years could an epiphenomenon of publication bias. Increased proportion of preterm neonates could be the co-product of improved neonatal intensive care. Although perforation rate have come down, it is yet to be conquered satisfactorily. Mortality is consistently zero since 1975. High mortality prior to that can be attributed to lack of broad spectrum antibiotics and primitive neonatal care. 

**Figure:**
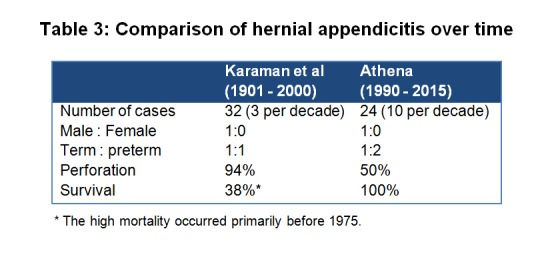
Table 3: Comparison of hernial appendicitis over time

**Conclusion**

 To conclude, all acute scrota need not be due to testicular torsion. Hernial appendicitis must be considered in the differential diagnosis. The affected newborn may deceptively look healthy except for the local swelling. Unexplained neonatal sepsis should also prompt a search in the groin. Scrotal erythema and warmth in a hernia should raise the suspicion of hernial appendicitis. 

## Footnotes

**Source of Support:** Nil

**Conflict of Interest:** The author is Editor of the journal. But he did not take part in the evaluation or decision making of this manuscript. The manuscript has been independently handled by two other editors.

